# Extracellular vesicles and pancreatitis: mechanisms, status and perspectives

**DOI:** 10.7150/ijbs.54858

**Published:** 2021-01-11

**Authors:** Yu-Chen Jia, Yi-Xuan Ding, Wen-Tong Mei, Yu-Ting Wang, Zhi Zheng, Yuan-Xu Qu, Kuo Liang, Jia Li, Feng Cao, Fei Li

**Affiliations:** 1 Department of General Surgery, Xuanwu Hospital, Capital Medical University, Beijing, China.; 2 Clinical Center for Acute Pancreatitis, Capital Medical University, Beijing, China.; 3 Capital Medical University, Beijing, China.

**Keywords:** pancreatitis, extracellular vesicles, exosomes, biomarkers

## Abstract

Comprehensive reviews and large population-based cohort studies have played an important role in the diagnosis and treatment of pancreatitis and its sequelae. The incidence and mortality of pancreatitis have been reduced significantly due to substantial advancements in the pathophysiological mechanisms and clinically effective treatments. The study of extracellular vesicles (EVs) has the potential to identify cell-to-cell communication in diseases such as pancreatitis. Exosomes are a subset of EVs with an average diameter of 50~150 nm. Their diverse and unique constituents include nucleic acids, proteins, and lipids, which can be transferred to trigger phenotypic changes of recipient cells. In recent years, many reports have indicated the role of EVs in pancreatitis, including acute pancreatitis, chronic pancreatitis and autoimmune pancreatitis, suggesting their potential influence on the development and progression of pancreatitis. Plasma exosomes of acute pancreatitis can effectively reach the alveolar cavity and activate alveolar macrophages to cause acute lung injury. Furthermore, upregulated exosomal miRNAs can be used as biomarkers for acute pancreatitis. Here, we summarized the current understanding of EVs in pancreatitis with an emphasis on their biological roles and their potential use as diagnostic biomarkers and therapeutic agents for this disease.

## Introduction

Pancreatitis refers to an inflammatory disorder of the pancreas, in which pancreatic enzymes damage pancreatic tissue, leading to acinar cell death, as well as local and systemic inflammation 1. Previous studies have shown that acute pancreatitis, recurrent acute pancreatitis, and chronic pancreatitis represent a continuum of disease progression. Per 100,000 people in the general population, the global incidence of acute pancreatitis is 33.74 cases per year and that of chronic pancreatitis is 9.62 cases per year 2. Similar to acute pancreatitis, chronic pancreatitis is most prevalent in middle-aged and older patients 3,4. However, the incidence of chronic pancreatitis was higher among men than women, although there was no significant difference between sexes for acute pancreatitis. The global transition rate data indicated that the transition from the first episode of acute pancreatitis to recurrent acute pancreatitis occurs in approximately 21% of cases and that from recurrent acute pancreatitis to chronic pancreatitis occurs in approximately 36% of cases 5. The global mortality rates of acute pancreatitis and chronic pancreatitis were 1.60 and 0.09 per 100,000 persons per year, respectively 2. Recently, clinical and experimental data have shed light on the pathophysiology of pancreatitis, indicating that premature intrapancreatic activation of digestive proteases is critical in the pathogenesis of pancreatitis 6. Furthermore, the progression and severity of pancreatitis may be influenced by dysregulated autophagy, which promotes the inflammatory response in the pancreas, leading to local and systemic inflammatory responses and multiorgan failure 7. Unfortunately, the sequelae and mortality of pancreatitis remain substantial. Concerted efforts by not only surgeons but also researchers should strive to reduce the incidence of pancreatitis and effectively improve the treatment of its sequelae 8-10.

EVs are cell-derived membranous structures that are present in biological fluids and are involved in physiological and pathological processes of inflammatory disease or cancer 11-15. EVs were initially regarded as membrane debris with no biological function 16. However, in 2007, exosomes were shown to transfer mRNAs and microRNAs to recipient cells, remained functional and changed the behavior of target cells 17. EVs exert their effects on fundamental biological processes by directly merging with the recipient cell plasma membrane and delivering their contents, including transcription factors, oncogenes, microRNAs and mRNAs, into recipient cells 18-20. In this manner, EVs participate in the pathophysiological process of disease, for example, stem cell therapy 21, tissue repair 22, immune surveillance 23, and tumor progression and metastasis 24,25. In addition, several studies have reported the potential applications of EVs in the diagnosis and treatment of disease based on their own characteristics.

Here, we report a comprehensive overview of the relationship between EVs and pancreatitis, with a special focus on their roles in pathogenesis and their potential clinical application as diagnostic biomarkers and therapeutic targets in pancreatitis. We also discuss the advantages and limitations among current studies and the need for further research. Finally, we discuss the prospects and applications of EVs in pancreatitis.

## EVs: clinical applications

EVs are regarded as a mechanism for intercellular communication, transferring proteins, lipids and genetic material between cells 26. Based on the current knowledge of their biogenesis by transmission electron microscopy, NanoSight analysis and other biochemical means, EVs can be broadly divided into two main categories: exosomes and microvesicles (MVs) 27,28. In addition, the pathophysiological roles of EVs are applied in the diagnosis of diseases including cancer and inflammatory diseases, especially in their potential treatments for therapeutic intervention 29,30.

### Diagnostic potential of EVs

The biomedical applications of EVs take advantage of their contents in the diagnosis and treatment of disease. The characteristic properties of EVs involve delivering functional cargos to diseased cells or EVs derived from diseased cells can affect normal cells; furthermore, EVs remain ill-defined in terms of their biological characteristics and functions 31. EVs contain a large number of extracellular and intracellular molecular components, which can be used as minimally invasive liquid biopsies for comprehensive, multiparameter disease diagnosis. EVs are diagnostic biomarkers for diseases include stroke 32, Alzheimer's disease 33, cardiovascular diseases 34 and cancer 35. Exosomal miRNAs are the most widely used diagnostic biomarkers, especially in cancer 36. Specific exosomal miRNAs may be diagnostic or prognostic markers in cancer. Furthermore, highly expressed oncogenic and tumor-suppressor miRNAs in exosomes may provide high diagnostic value due to their differential expression between cancer cells and normal cells, especially in the early diagnosis of diseases 37. Similarly, exosomal proteins also have diagnostic potential for diseases. Several studies have reported the utility of glypican-1 (GPC1)-positive exosomes in the diagnosis of pancreatic cancer 38-40. GPC1 is specifically enriched in pancreatic patient serum-derived exosomes, distinguishing chronic patients and healthy people from patients with early- or late-stage pancreatic cancer. Thus, the multicomponent and combinatorial nature of exosomal proteins and miRNAs could potentially enhance the specificity and sensitivity of cancer diagnosis and prognostic evaluation. Therefore, EVs can be used as biomarkers for disease diagnosis because disease-generating exosomes can reflect disease-specific changes.

### Therapeutic potential of EVs

According to the characteristics of EVs that can contain DNA, RNA and proteins, exosomes by themselves or as vehicles for drug delivery have therapeutic potential in diseases 41-43. Exosomes, as natural endogenous drugs, have obvious advantages in delivering functional cargo to cells. Compared with liposomes, exosomes are widely distributed in body fluids with low immunogenicity and minimal immune clearance. Furthermore, their phospholipid bilayer effectively protects the loaded drugs, making them stable in the blood 44. Several studies reported that exosomes from mesenchymal stem cells (MSCs) or dendritic cells inhibit disease progression by transporting siRNAs 45, miRNAs 46,47, and chemotherapy drugs 48-50. In addition, ligand-modified exosomes may be used to enhance their targeting ability to specific cell types 51,52. For example, previous studies reported that the integrin-specific recognition peptide RGD was applied for exosome membrane modification to enhance the exosome targeting capability 53. Tian et al. reported that exosomes derived from immature dendritic cells deliver doxorubicin to human breast cancer cells, inhibiting tumor progression without obvious toxicity 54. Together, these clinical and experimental data contribute to the development of exosomes as therapeutic vesicles.

## EVs and AP

### EVs in the pathogenesis of AP

Previous studies have reported that the pathogenesis of acute pancreatitis includes calcium signaling 55, premature trypsinogen activation 56, autophagy 57, endoplasmic reticulum stress, the unfolded protein response 58, intraductal fluid stasis 59, immune system 60, genetic mutations 61, unsaturated fatty acids 62 and mesenteric lymph 63, which mainly lead to trypsinogen activation and injury of acinar cells. The most common and earliest organ dysfunction of AP-associated complications is acute lung injury (ALI), accounting for approximately 10-25% of the incidence and 60% of the mortality 63-65. Underlying mechanisms of AP-associated ALI are complex and poorly understood, although recent perspectives have indicated that pancreatic phospholipase A2, proinflammatory cytokines, neutrophil sequestration and bacterial translocation are involved in the mechanisms of AP and ALI 66-68.

**Figure 1:**
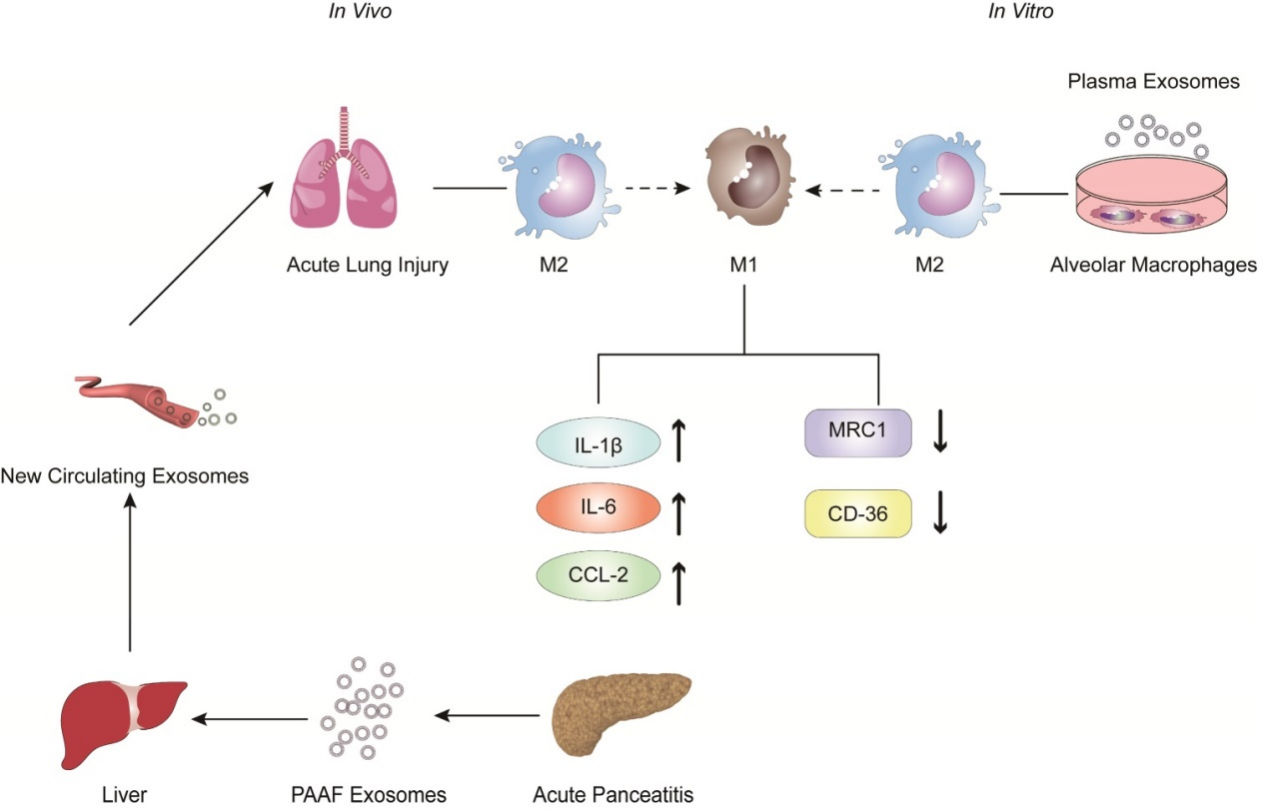
The role of extracellular vesicles in the mechanism of AP-related alveolar macrophage activation. The figure shows that *in vitro* experiments (left) revealed that part of the PAAF exosomes released from the pancreas during AP entered the liver directly through the portal system, and most of them were retained in liver tissue. During AP, the formation of new circulating exosomes from the liver reached the alveoli and activated the alveolar macrophages from the M2 phenotype to the pro-inflammatory M1 phenotype, leading to significantly increased expression of cytokines IL-1, IL-6 and chemokine CCL2, while the expression of MRC1 and CD36 was decreased. *In vivo* experiments (right) showed that plasma exosomes from AP promoted the activation of alveolar macrophages from the M2 phenotype to the pro-inflammatory M1 phenotype, and resulted in significantly increased expression of the inflammatory cytokines IL-1 and the chemokine CCL2 in alveolar macrophages.

In recent years, the role of exosomes has been gradually clarified in the pathogenesis and treatment of inflammatory diseases, especially in AP 69-75. Bonjoch et al. illustrated that the increased plasma exosomes of acute pancreatitis effectively reach the alveolar cavity and activate alveolar macrophages in an experimental rat model of taurocholate-induced acute pancreatitis 76. Moreover, *in vitro* experiments showed that plasma exosomes activate alveolar macrophages from the M2 phenotype to a proinflammatory M1 phenotype, concurrent with significantly increased expression of the M1 marker cytokines IL-1β and IL-6 and the chemokine CCL-2 and decreased expression of the M2 markers MRC1 and CD36. In addition, mass spectrometry-driven proteomic analysis of plasma exosomes indicated that the 33 significantly differentially expressed proteins were mainly derived from liver and immune cells; however, the expression of protein derived from the pancreas was downregulated. Thus, proteomic analysis suggested that the most likely origin of plasma exosomes could be the liver instead of the pancreas. Tracking analysis and histological analysis revealed that the liver retains almost 75% of exosomes from pancreatitis-associated ascitic fluid (PAAF). Furthermore, exosomes filtered by the liver changed not only in number but also in protein content. These results indicated that the liver could be generating and releasing new exosomes during AP, which could reach the alveoli and activate alveolar macrophages to a proinflammatory phenotype (Figure 1).

**Figure 2:**
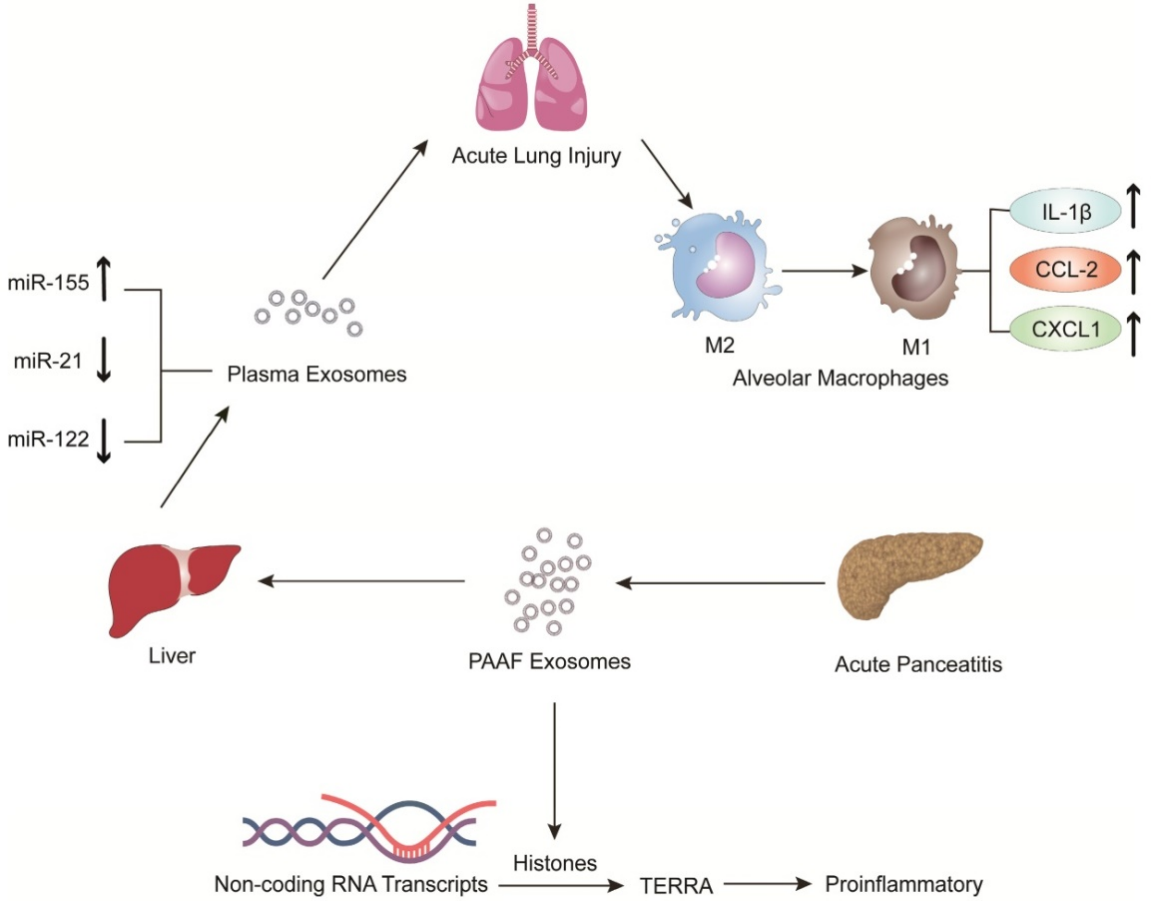
The expression of pro-inflammatory miR-155 in plasma exosomes produced during AP was significantly increased, while the expressions of anti-inflammatory miR-122 and miR-21 were decreased. The arrival of plasma exosomes to the alveoli and by activating alveolar macrophages leads to increased expression of the inflammatory cytokines IL-1 and the chemokines CCL2 and CXCL1, thus exacerbating AP-related lung injury. In addition, PAAF exosomes produced during AP contain more histones, which induce inflammation by regulating the transcription of non-coding RNA to produce TERRA.

Jiménez-Alesanco et al. performed further experiments to show that the liver could be the source of plasma exosomes that activate the inflammatory response in the lung, rather than the pancreas, during AP 77. These researchers provided evidence that plasma exosomes and PAAF exosomes differ in microRNA (miRNA) content, protein, distribution and physiological effects. Exosomal miRNA analysis revealed that plasma exosomes contained high expression of miR-155 and low expression of miR-122 and miR-21; however, the expression of these miRNAs in PAAF exosomes was similar to that in the control group. Previous studies have shown that miR-155 has a proinflammatory role that can promote M1 polarization of macrophages 78. In contrast, miR-122, which is mainly produced by the liver, plays an anti-inflammatory role 79,80. Therefore, the results suggested that proinflammatory miR-155 expression was significantly upregulated, concurrent with a significant decrease in anti-inflammatory miR-21 and miR-122 expression in plasma exosomes, which could play a proinflammatory response by activating macrophages and promoting the release of inflammatory cytokines. Moreover, proteomic analysis revealed that the proteins of plasma exosomes were mainly from the liver; however, only two specific pancreatic proteins were detected. PAAF exosomes contained high levels of pancreatic enzymes, which confirmed their pancreatic origin. However, histones and ribosomal proteins were more enriched in PAAF exosomes but not in plasma exosomes. Furthermore, histone proteins produce telomeric repeat-containing RNA (TERRA) by regulating noncoding RNA transcripts, which are carried by exosomes from damaged cells to induce an inflammatory response 81,82. They also evaluated the different effects of plasma exosomes and PAAF exosomes on alveolar macrophages. The results showed that plasma exosomes significantly increased the expression of the inflammatory cytokine IL-1β and chemokines CCL2 and CXCL1 in alveolar macrophages; however, the increase in inflammatory factors was not statistically significant in PAAF exosomes compared with that in the control group. The results indicated that these highly expressed miRNAs and proteins could be targeted for designed therapeutic drugs for the treatment of AP (Figure 2).

**Figure 3:**
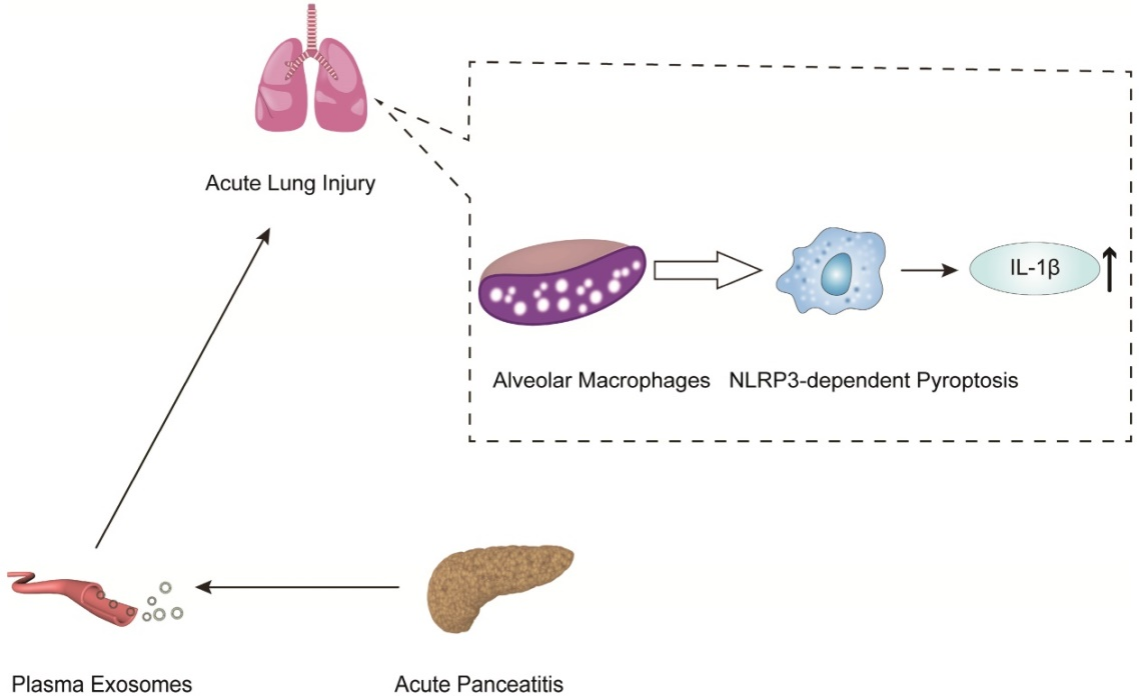
AP-generated plasma exosomes activate NLRP3 inflammasomes in alveolar macrophages and induce NLRP3-dependent pyroptosis, leading to apoptosis in alveolar macrophages and increased expression of the inflammatory cytokine IL-1.

In another mechanistic study of pancreatitis-associated ALI, Wu et al. indicated that plasma exosomes triggered NOD-like receptor protein 3 (NLRP3)-dependent pyroptosis in alveolar macrophages, which induced AP-associated ALI 83 (Figure 3). The present work revealed that plasma exosomes stimulated alveolar macrophages to activate the NLRP3 inflammasome, released IL-1β and induced pyroptosis, suggesting that the plasma exosome-mediated NLRP3 pathway is a potential therapeutic target for the treatment of ALI during AP (Figure 3).

### EVs in the diagnosis of AP

There are many studies on exosomal miRNAs as a diagnostic marker of inflammatory disease, including alcoholic hepatitis 84, inflammatory liver diseases 85, diabetes mellitus 86, liver disease 87, and Parkinson's disease 88. However, there are only a few studies on exosomal miRNAs as diagnostic biomarkers for AP 89. Zhao et al. indicated that 115 differentially expressed exosomal miRNAs of the pancreatic acinar cell line AR42J were identified by a miRNA microarray. Among the differentially expressed miRNAs, 30 were upregulated and 85 were downregulated. Therefore, these 30 upregulated miRNAs may be used as biomarkers for AP. However, the results of this study are only derived from *in vitro* experiments and have not been verified by *in vivo* experiments and human samples. It is not yet known whether there are any types of interference, such as differential expression and exosome rupture. Furthermore, target genes of the identified miRNAs were predicted using TargetScan and analyzed by KEGG pathway analysis. The pathways included cell adhesion molecules (CAMs), glycerophospholipid metabolism, the Wnt signaling pathway, the MAPK signaling pathway and the Hedgehog signaling pathway. After further analysis and verification, the target genes regulated macrophage and NFκB activation through the TRAF6-TAB2-TAK1-NIK/IKK-NFκB pathway, which is one of the MAPK signaling pathways. Therefore, the present study provides new ways to alleviate pancreatitis-associated macrophage activation and potential diagnostic exosomal biomarkers for AP.

### EVs in the treatment of AP

**Figure 4:**
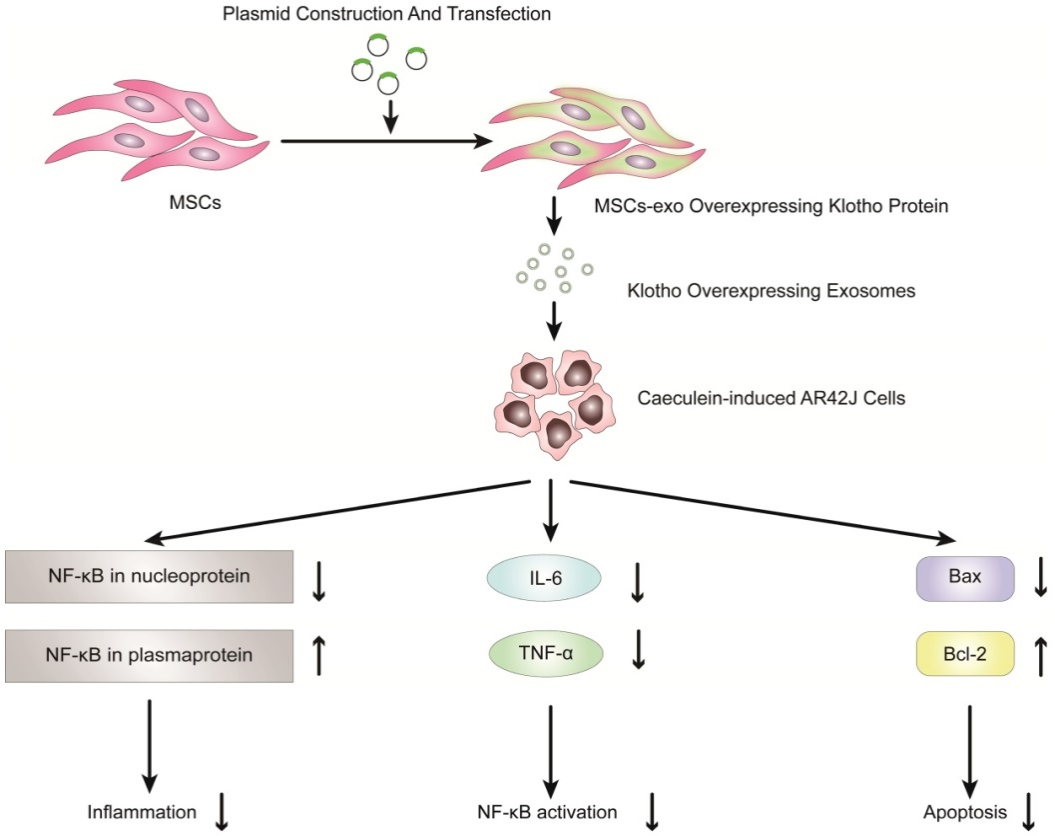
Overexpression of Klotho protein in exosomes from genetically engineered mesenchymal stem cells can reduce the inflammatory response of pancreatic acinar cells (AR42J cells) in the model of acute pancreatitis induced by caerulein.

Previous studies have shown that exosomes derived from mesenchymal stem cells (MSCs) can be used to reduce inflammatory responses and treat inflammatory diseases 90-100. Therefore, MSC-derived exosomes have potential clinical value in the treatment of inflammatory diseases, especially pancreatitis. Wang et al. reported that exosomes derived from mesenchymal stem cells that overexpress Klotho attenuated the severity of pancreatic inflammation in caerulein-stimulated AR42J cells 101. Klotho, which is expressed in pancreases, is essential for digestive enzyme secretion from pancreatic acinar cells 102. In this study, exosomes derived from MSCs that overexpressed Klotho (MSCs-exo Klotho) decreased the expression of IL-6 and TNF-α compared to that of the control group. Furthermore, the expression of Bax and NF-kB in nucleoproteins was significantly downregulated in the MSC-exo Klotho group, concurrent with a significant increase in the expression of Bcl-2 and NF-kB in plasma proteins. In conclusion, these results showed that MSC-exo Klotho alleviated inflammation and apoptosis in AP and that Klotho could be a potential targeted therapy for clinical treatment in AP (Figure 4).

## EVs and CP

### EVs in the pathogenesis of CP

**Figure 5:**
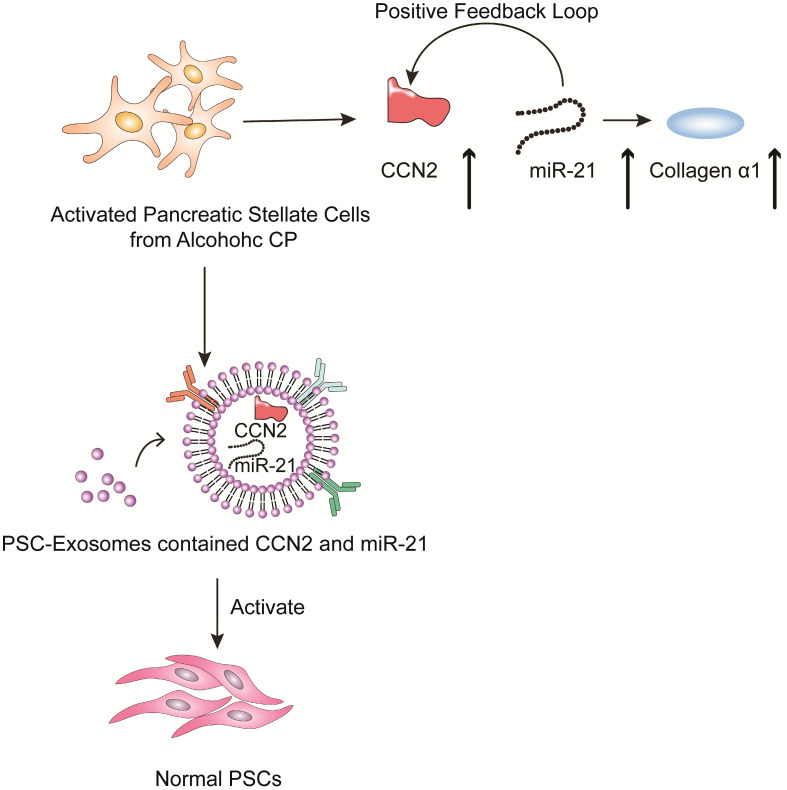
Role of extracellular vesicles in CP - associated pancreatic fibrosis. The figure shows that in activated PSC in alcoholic chronic pancreatitis, up-regulated CCN2 and miR-21 constitute a positive feedback pathway that promotes collagen α1 production. In addition, exosomes produced by activated PSCs contained CCN2 and miR-21, and these exosomes could activate more PSCs and produce more exosomes and collagen α1.

Chronic pancreatitis is a chronic inflammatory disease that is characterized by fibrosis and inflammation of the pancreas, with genetic, environmental, and other risk factors 103-107. The pathophysiological processes of CP mainly involve acinar cell injury 108,109, inflammation 110 and fibrosis by activated pancreatic stellate cells 111. Studies have shown that activated pancreatic stellate cells (PSCs) are considered a promoter of pancreatic fibrosis, which is a crucial hallmark of CP 112-115. Activated PSCs are the main producers of connective tissue growth factor (CCN2), which plays an important role in driving fibrogenic pathways to stimulate extracellular matrix collagen production. Charrier et al. found that the expression of CCN2 and miR-21 is upregulated in PSCs 116. CCN2 not only drives collagen expression but also stimulates the expression of miR-21, which can itself increase CCN2 expression. Thus, upregulated CCN2 and miR-21 are components of a positive feedback loop that may be a mechanism for enhanced collagen production in CP. Additionally; the study indicated that the exosomes derived from activated PSCs contain CCN2 and miR-21, which can be shuttled to activate normal PSCs. Therefore, inhibiting exosome secretion and the expression of CNN2 and miR-21 can reduce the inflammatory response caused by PSC activation in the development of CP (Figure 5).

### EVs in the diagnosis of CP

In the diagnosis of chronic pancreatitis, effective diagnostic biomarkers are still lacking.

At present, no study has indicated that exosomes can be used as diagnostic biomarkers to distinguish chronic pancreatitis from normal conditions. Pancreatic ductal adenocarcinoma (PDAC) is sometimes difficult to distinguish from chronic pancreatitis in the early clinical diagnosis. Furthermore, CP may be misdiagnosed as PDAC, leading to unnecessary pancreatic resection. Therefore, accurate early diagnosis and clear differentiation between PDAC and CP are crucial for patients 104,106.

In addition, several studies have shown that exosomal miRNAs can distinguish patients with chronic pancreatitis from those with PDAC. Lai et al. 117 found that high expression of exosomal miR-10b, miR-20a, miR-21, miR-30c, miR-106b and miR-181a and low expression of exosomal miR-let7a can effectively differentiate patients with PDAC from those with CP. Moreover, after resection, the high expression of exosomal miR-10b, miR-20a, miR-21, miR-30c, and miR-106b decreased to normal values. Nakamura et al. 118 reported that they used exosomal miRNAs from pancreatic juice to distinguish patients with PDAC from those with CP. Quantitative real-time reverse transcription polymerase chain reaction (qRT-PCR) showed that the expression levels of exosomal miR-21 and miR-155 were significantly higher in the PDAC patients than in the CP patients. However, there were no significant differences observed in the expression levels of free miR-21 and free miR-155 in PDAC and CP patients. Furthermore, the AUC values of exosomal miR-21 and miR-155 levels were significantly higher than those for the serum CA19-9 levels. Therefore, exosomal miRNAs may be useful and stable biomarkers for distinguishing patients with chronic pancreatitis from those with PDAC.

Reese et al. used qRT-PCR to show that the expression of miR-200b and miR-200c was significantly downregulated in serum exosomes of PDAC patients compared to healthy controls (HCs) and patients with CP. Moreover, the expression of exosomal miR-125b was significantly upregulated in patients with CP compared to those with HC, and the expression of exosomal miR-148a was significantly upregulated in patients with CP compared to PDAC patients 119. Therefore, exosomal miR-125b and miR-148a can be used as specific diagnostic biomarkers to distinguish CP patients from patients with HC and PDAC, respectively. Similarly, other studies have shown that exosomal miR-10b and miR‑23b‑3p can also distinguish CP from PDAC 120,121.

Furthermore, several studies have shown that exosomal DNA can distinguish patients with chronic pancreatitis from those with PDAC. Yang et al. 122 indicated that circulating exosomal double stranded genomic DNA derived from PDAC patients enabled the detection of prevalent KRAS and TP53 mutations. Digital PCR of exosomal DNA identified KRAS mutations in 29 of 48 (39.6%) cases and TP53 mutations in 2 of 48 (4.2%) cases in PDAC patients. Moreover, they found that 3 of 7 (42.8%) IPMN patients harbored the KRAS mutation, and one of these patients also coharbored the TP53 mutation. For CP patients, the KRAS mutation was found in 5 of 9 (55.6%) cases; however, none had the TP53 mutation. In addition, 5 of 12 (41.7%) patients with other diseases, such as autoimmune pancreatitis, common bile duct cancer, pancreatic cystadenoma, and pancreatic neuroendocrine tumor, harbored the KRAS mutation, and only 1 had the TP53 mutation. In healthy subjects, the KRAS mutation was observed in 3 of 114 (2.6%) cases, and none had the TP53 mutation. Therefore, this study demonstrates that circulating exosomal KRAS and TP53 mutations can be used to distinguish healthy subjects from those with PDAC, PC and other diseases.

Several studies have applied exosomal miRNAs and DNA as diagnostic biomarkers of disease; however, whether the concentrations and diameter of EVs could discriminate malignant and benign disease has not been determined. Severino et al. 123 used the concentrations of EVs to discriminate malignant from nonmalignant CBD stenoses. They collected EVs derived from bile and blood samples and assessed them by nanoparticle tracking analyses (NTA). In bile samples, the concentration of EVs ranged between 1.78×10^12^ and 1.31×10^16^ nanoparticles/L, with an overall median value of 6.66×10^14^ nanoparticles/L. In the PDAC group vs the biliary stones group, the median concentration of EVs was 2.41×10^15^ vs 1.60×10^14^ nanoparticles/L. Furthermore, a threshold of 9.46×10^14^ nanoparticles/L in bile samples distinguished patients with PDAC from those with biliary stones. In the PDAC group vs the CP group, the median concentration of EVs was 4.00×10^15^ vs 1.26×10^14^ nanoparticles/L. The threshold of 9.46×10^14^ nanoparticles/L discriminated PDAC from CP with 100% accuracy. In serum samples, the concentration of EVs was significantly lower than that in bile samples, with an overall median of 2.67×10^13^ nanoparticles/L. The median concentration of EVs in the PDAC group vs the biliary stone group was 3.55×10^14^ vs 1.74×10^13^ nanoparticles/L. In the PDAC group vs the CP group, the median concentration of EVs was 4.64×10^13^ vs 7.58×10^12^ nanoparticles/L. In addition, the average diameter of EVs in the PDAC group was 277.8 nm; however, the average diameter of EVs in the CP group was 169.9 nm. Furthermore, EVs derived from bile have larger sizes and contain more proteins in the malignant vs nonmalignant group. Thus, the concentrations and diameters of EVs could be used to discriminate malignant from benign disease with optimal accuracy.

## EVs and AIP

Autoimmune pancreatitis (AIP) is a special form of CP that has a pivotal role in inducing fibroinflammatory disorders of the pancreas 124,125. The diagnosis and treatment of AIP have not achieved satisfactory clinical effects. Nakamaru et al. reported that the expression of miR-21 was significantly upregulated in extracellular vesicles derived from the serum of patients with type 1 autoimmune pancreatitis 126. This study included 27 patients with type 1 AIP, 23 patients with chronic pancreatitis and 23 healthy controls (HCs). Microarray analysis showed 165 differentially expressed miRNAs in patients with type 1 AIP. Furthermore, 132 miRNAs were upregulated and 33 were downregulated in type 1 AIP patients compared with HCs. Among these results, the expression levels of miR-659-3p, miR-27a-3p, miR-99a-5p, miR-21-5p, miR-205-5p, miR-100-5p, miR-29c-3p, and miR-126b-1-3p were significantly upregulated, concurrent with a significant decrease in miR-4252 and miR-5004-1-5p expression relative to that of the HCs. Quantitative evaluation of EV miRNA expression levels by RT-PCR showed that only the expression level of miR-21-5p was significantly higher in type 1 AIP patients than in HCs. Furthermore, the results of *in situ* hybridization (ISH) of resected specimens of type 1 AIP patients showed that the expression of miR-21 in pancreatic duct epithelium was similar between type 1 AIP patients and HCs. However, miR-21 was highly expressed in pancreatic acinar cells in type 1 AIP patients compared to HCs. Therefore, this study demonstrated that miR-21 in EVs derived from AIP patients' serum could be used as a diagnostic marker to distinguish AIP from healthy people.

## Shortcoming and perspectives

The study of EVs has the potential to identify cellular and molecular communication and value in the diagnosis and treatment of diseases 26,31. Due to their diverse and unique contents, such as nucleic acids, proteins, lipids, and metabolites, EVs not only can reflect their origin cells but can also be used as a diagnostic marker for disease. In addition, EVs protect their contents through their stable membrane structure and serve as an effective carrier for drug delivery in the therapeutics of cancer and inflammatory diseases 15,36,127.

In recent years, EV research has focused on the classification of EVs, isolation methods, and their functions in disease diagnosis, progression and therapy 30,128-130. Despite the increase in different isolation methods of EVs, there are still no uniform and standardized methods available for the purification and isolation of EVs 131,132. Therefore, it remains unclear whether different isolation methods of EVs may lead to different results 26. Currently, there is a need to establish standardized methods of sample collection, storage, and application to minimize the influence of the complexity and heterogeneity of EVs 133. In addition, the conventional isolation methods of exosomes in blood, such as ultracentrifugation, cannot completely remove lipoprotein, which is similar in size and density to EVs 134. However, the volume and time of blood collection, handling, storage condition and application of anticoagulants all impact the isolation of EVs 135. For the isolation of EVs from cultured cells, it is recommended to use serum-free medium or EV-free serum in cell culture medium 136. For clinical blood sample collection, it is important to minimize the influence of the activation and release of platelet and red blood cell-derived EVs and the contamination of cell debris. A number of studies have shown that -80°C and minimized freeze-thaw cycles are the optimum conditions for the storage of EVs based on size, composition, and functionality. An unfavorable temperature and increased freeze-thaw cycles can cause EV aggregation and lysis, leading to an increase in size, a reduction in counting and a loss of content. The current methods of EV isolation mainly include ultracentrifugation, size-exclusion chromatography, filtration, commercial reagents, microfluidics, asymmetric flow field-flow fractionation, and nanoflow cytometry 133. Diverse methods have their advantages and disadvantages. Moreover, high-efficiency isolation of EVs is needed to eliminate protein contamination and increase purity for clinical application of EVs.

In the diagnosis of AP, there are only a few studies on exosomal biomarkers for AP. Zhao et al. indicated that 30 exosomal miRNAs were upregulated in pancreatic acinar AR42J cells and could be used as biomarkers for AP. However, the results of this study are only derived from *in vitro* experiments and have not been verified by *in vivo* experiments and human samples 89. Therefore, for an EV diagnostic biomarker of AP, large samples and multicenter clinical studies are needed. In the diagnosis of CP, no literature has indicated that EVs can be used as diagnostic biomarkers to distinguish CP patients from healthy people. However, PDAC is sometimes difficult to distinguish from CP in the early clinical diagnosis, leading to unnecessary pancreatic resection 104,106. Therefore, Lai et al. 117 found that high expression of exosomal miR-10b, miR-20a, miR-21, miR-30c, miR-106b and miR-181a can effectively differentiate patients with PDAC from those with CP. After resection, the high expression of these miRNAs decreased to normal values. Moreover, Nakamura et al. 118 reported that the expression of exosomal miR-21 and miR-155 from pancreatic juice was significantly higher in PDAC patients than in CP patients. Compared with those of serum CA19-9 levels, the AUC values of exosomal miR-21 and miR-155 levels were significantly higher. Therefore, exosomal miRNAs may be useful and stable biomarkers for distinguishing patients with CP from those with PDAC. Furthermore, several studies have shown that exosomal DNA can also distinguish patients with CP from those with PDAC and healthy subjects. Yang et al. 122 indicated that exosomal DNA identified KRAS mutations in 29 of 48 (39.6%) cases and TP53 mutations in 2 of 48 (4.2%) cases in PDAC patients. For CP patients, the KRAS mutation was found in 5 of 9 (55.6%) cases; however, none had the TP53 mutation. In healthy subjects, the KRAS mutation was observed in 3 of 114 (2.6%) individuals, and none had the TP53 mutation. Therefore, the study indicates that circulating exosomal KRAS and TP53 mutations can be used to distinguish patients with CP from those with PDAC and healthy subjects.

In recent years, compared with exosomal miRNAs and DNA as diagnostic biomarkers of disease, the concentrations and diameters of EVs could discriminate PDAC and CP patients 123. In bile samples, the median concentration of EVs was 4.00×10^15^ vs 1.26×10^14^ nanoparticles/L in the PDAC group vs the CP group. In serum samples, the median concentration of EVs was 4.64×10^13^ vs 7.58×10^12^ nanoparticles/L in the PDAC group vs the CP group. In addition, the average diameter of EVs in the PDAC group was 277.8 nm; however, the average diameter of EVs in the CP group was 169.9 nm. Thus, EVs derived from bile have larger sizes and contain more proteins in the PDAC vs CP group.

In the diagnosis of AIP, Nakamaru et al. reported that the expression of miR-21 was significantly upregulated in extracellular vesicles derived from serum from patients with type 1 autoimmune pancreatitis 126. This study included 27 patients with type 1 AIP, 23 patients with chronic pancreatitis and 23 healthy controls (HCs). Microarray analysis and RT-PCR showed that the expression level of miR-21-5p was significantly higher in type 1 AIP patients than in HCs. Therefore, miR-21 of EVs derived from AIP patients' serum could be used as a diagnostic marker to distinguish AIP patients from healthy people. At present, there are no reports on potential therapeutic application of EVs in the treatment of AIP. However, it has been reported that EVs have potential therapeutic effect in other autoimmune diseases, such as type 1 diabetes mellitus, multiple sclerosis, systemic lupus erythematosus 134. Research has been suggested that mesenchymal stem cells-derived exosomes might protect the pancreatic islets of patients with Type 1 diabetes by immunomodulatory effect to slow disease progression 135. Similarly, Multiple sclerosis (MS) is a T cell-mediated autoimmune disease, which underlying mechanisms are unclear. Kimura have showed that MS derived exosomal let-7i regulates MS pathogenesis by blocking the insulin like growth factor 1 receptor and transforming growth factor beta receptor 1 pathway 136. Therefore, more studies are needed to further investigate the mechanism and treatment of EVs in AIP.

In the treatment of AP, previous studies have shown that exosomes derived from MSCs can be used to reduce inflammatory responses and treat inflammatory diseases 90-100. Therefore, Wang et al. reported that exosomes derived from mesenchymal stem cells that overexpress Klotho attenuated the severity of pancreatic inflammation in caerulein-stimulated AR42J cells 101. In this study, exosomes derived from MSCs that overexpressed Klotho decreased the expression of IL-6 and TNF-α compared to the control group. In conclusion, these results showed that MSC-exo Klotho alleviated inflammation and apoptosis in AP and that Klotho could be a potential targeted therapy for clinical treatment in AP. However, EVs have not been applied for drug delivery in the treatment of AP. The application of drug-loaded EVs can effectively improve the targeting ability of drugs. In addition, compared with liposomes, EVs have an advantage in the application of drug delivery for targeted treatment.

## Conclusion

In this review, we report a comprehensive overview of the relationship between EVs and pancreatitis, with a special focus on their roles in pathogenesis and their potential clinical applications as diagnostic biomarkers and therapeutic targets in pancreatitis. We have discussed EV isolation and application of clinical-grade EVs and the advantages and limitations of current studies, as well as the need for further research. EVs are regarded as a mechanism for intercellular communication, transferring proteins, lipids and genetic material in pancreatitis.

EVs are involved in the pathogenesis of pancreatitis and are used as diagnostic markers for pancreatitis and have clear potential as treatment targets in pancreatitis. However, there are still no uniform and standardized methods available for the purification and isolation of EVs. Therefore, it remains unclear whether different isolation methods of EVs may lead to different results. Currently, there is a need to establish standardized methods of sample collection, storage, and application to minimize the influence of the complexity and heterogeneity of EVs. EVs have not been applied drug delivery in the treatment of AP. Therefore, the clinical application of EVs in the diagnosis and treatment of pancreatitis is promising, and additional extensive research is required before clinical application.

## Figures and Tables

**Table 1:** The role of EVs in pancreatitis

Pancreatitis	Category	Source of EVs	Related molecules	Effects	References
AP	Pathogenesis	Mice plasma	IL-1β, IL-6, CCL-2, MRC1, CD36	The increased plasma exosomes of acute pancreatitis effectively reach the alveolar cavity and activate alveolar macrophages in AP.	76
		Mice plasma, Mice PAAF	miR-155, miR-122, miR-21, TERRA, IL-1β, CCL2, CXCL1	The liver could be the source of plasma exosomes that activate the inflammatory response in the lung, rather than the pancreas, during AP.	77
		Mice plasma	NLRP3,IL-1β	Plasma exosomes triggered NOD-like receptor protein 3 (NLRP3)-dependent pyroptosis in alveolar macrophages, which induced AP-associated ALI.	83
	Diagnosis	AR42J cell	miRNAs	Upregulated extracellular vesicle miRNAs in TRAF6-TAB2-TAK1-NIK/IKK-NFκB pathway may be used as biomarkers for AP.	89
	Treatment	MSCs	Klotho, IL-6, TNF-α,Bax, Bcl-2	Exosomes derived from mesenchymal stem cells that overexpress Klotho attenuated the severity of pancreatic inflammation in caerulein-stimulated AR42J cells.	101
		Mice plasma	NLRP3,IL-1β	Exosome-mediated NLRP3 pathway is a potential therapeutic target for the treatment of ALI during AP.	83
CP	Pathogenesis	PSC	CCN2,miR-21	CCN2 up-regulation in PSC is associated with increased expression of miR-21 which, in turn, is able to stimulate CCN2 expression further via a positive feedback loop. Additionally miR-21 and CCN2 were identified in PSC-derived exosomes which effected their delivery to other PSC.	116
	Diagnosis	Human plasma	miR-10b, miR-20a, miR-21, miR-30c, miR-106b, miR-181a, miR-let7a	Clear differentiation between PDAC and CP.	117
		Human Pancreatic juice	miR-21, miR-155	Clear differentiation between PDAC and CP.	118
		Human serum	miR-125b, miR-148a	Clear differentiation between PDAC and CP.	119
		Human serum	miR-10b,miR‑23b‑3p	Clear differentiation between PC and CP.	120,121
		Human serum	DNA	Circulating exosomal KRAS and TP53 mutations can be used to distinguish healthy subjects from those with PDAC and PC.	122
		Human bile, Human serum	EVs' concentrations	Discriminate malignant from nonmalignant CBD stenoses.	123
	Treatment	PSC	CCN2, miR-21	Inhibiting exosome secretion and the expression of CNN2 and miR-21 may reduce the inflammatory response caused by PSC activation in the development of CP.	116
AIP	Pathogenesis	Human serum	miR-21	Diagnostic marker to distinguish AIP from healthy people.	126

## Abbreviations

EVs: Extracellular vesicles
AP: Acute pancreatitis
CP: Chronic pancreatitis
AIP: Autoimmune pancreatitis
MVs: Exosomes and microvesicles
GPC1: Glypican-1
MSCs: Mesenchymal stem cells
ALI: Acute lung injury
PAAF: pancreatitis-associated ascitic fluid
miRNAs: microRNAs
TERRA: Telomeric repeat-containing RNA
NLRP3: NOD-like receptor protein 3
CAMs: Cell adhesion molecules
MSCs: Mesenchymal stem cells
MSCs-exo klotho: Exosomes derived from MSCs which overexpressed klotho
PSCs: Pancreatic stellate cells
CCN2: Connective tissue growth factor
PDAC: Pancreatic ductal adenocarcinoma
qRT-PCR: Quantitative real-time reverse transcription polymerase chain reaction
HC: Healthy control
IPMN: Intraductal papillary mucinous neoplasm
NTA: Nanoparticle tracking analyses
HCs: Healthy controls
ISH: *In situ* hybridization
